# Forecasting Individual Aging Trajectories and Survival with an Interpretable Network Model

**DOI:** 10.1093/geroni/igaa057.3387

**Published:** 2020-12-16

**Authors:** Spencer Farrell, Arnold Mitnitski, Kenneth Rockwood, Andrew Rutenberg

**Affiliations:** Dalhousie University, Halifax, Nova Scotia, Canada

## Abstract

We have built a computational model of individual aging trajectories of health and survival, containing physical, functional, and biological variables, conditioned on demographic, lifestyle, and medical background information. We combine techniques of modern machine learning with a network approach, where the health variables are coupled by an interaction network within a stochastic dynamical system. The resulting model is scalable to large longitudinal data sets, is predictive of individual high-dimensional health trajectories and survival, and infers an interpretable network of interactions between the health variables. The interaction network gives us the ability to identify which interactions between variables are used by the model, demonstrating that realistic physiological connections are inferred. We use English Longitudinal Study of Aging (ELSA) data to train our model and show that it performs better than standard linear models for health outcomes and survival, while also revealing the relevant interactions. Our model can be used to generate synthetic individuals that age realistically from input data at baseline, as well as the ability to probe future aging outcomes given an arbitrary initial health state.

